# Actin in Action: Imaging Approaches to Study Cytoskeleton Structure and Function

**DOI:** 10.3390/cells2040715

**Published:** 2013-11-14

**Authors:** Katey K. McKayed, Jeremy C. Simpson

**Affiliations:** School of Biology and Environmental Science & Conway Institute of Biomolecular and Biomedical Research, University College Dublin, Dublin 4, Ireland; E-Mail: katey.mckayed@ucd.ie

**Keywords:** cytoskeleton, actin, focal adhesions, fluorescence imaging, live cell imaging, high content screening and analysis

## Abstract

The cytoskeleton plays several fundamental roles in the cell, including organizing the spatial arrangement of subcellular organelles, regulating cell dynamics and motility, providing a platform for interaction with neighboring cells, and ultimately defining overall cell shape. Fluorescence imaging has proved to be vital in furthering our understanding of the cytoskeleton, and is now a mainstay technique used widely by cell biologists. In this review we provide an introduction to various imaging modalities used to study focal adhesions and the actin cytoskeleton, and using specific examples we highlight a number of recent studies in animal cells that have advanced our knowledge of cytoskeletal behavior.

## 1. Introduction

A key tenet of cell biology lies in the intimate link between structure and function. At the macro scale, specific cell morphology can be related to the function of that cell within a tissue; and similarly at the molecular scale, precise organelle positioning and dynamics are also critical for their purpose within the cell. The visualization of cells and their subcellular structures not only enhances our understanding of form, but quantification of visual data can also improve our knowledge of function. Cell shape and the manner in which cells interact with the surrounding environment influence the regulation of many cellular processes, including proliferation, differentiation, motility and apoptosis [1,2,3,4]. In this regard, the cytoskeleton plays a central role, and its dynamic nature allows for rapid rearrangement of its components in order to fulfill the cell’s morphological requirements at particular time points in its life cycle. Furthermore, the cytoskeleton and its associated proteins participate in a wide variety of signal transduction pathways, which can ultimately dictate cell fate. The cytoskeleton is composed of three main components; microtubules, intermediate filaments and the actin network, all of which have the ability to resist deformation, reorganize in response to externally applied forces or stimuli, and maintain the spatial relationships between subcellular compartments [reviewed in [5]].

**Figure 1:**
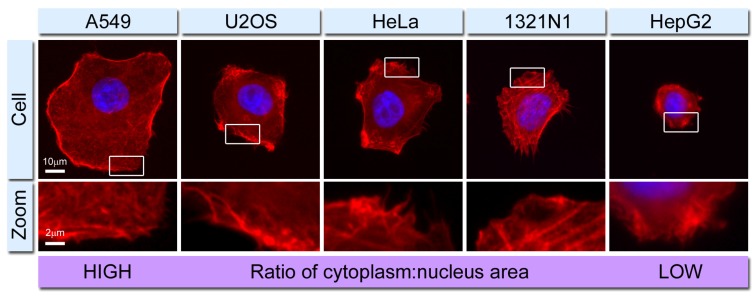
Fluorescence imaging of human cell lines used to study actin cytoskeleton function. The actin network (red) is labeled with fluorescently-conjugated phalloidin, and the nuclei (blue) are labeled with Hoechst 33342. Actin is seen to have a very complex localization pattern that differs across the various cell lines. In some cells it is highly concentrated close to the plasma membrane and in filopodial protrusions, in other cells stress fibers can be seen, and in some instances small punctate actin accumulations are present. The figure also shows the differing ratios of cytoplasm-to-nucleus area that exist across different cell lines, thereby highlighting the importance of making the most suitable choice of cell for actin imaging experiments.

Microtubules are highly dynamic structures composed of α- and β-tubulin heterodimers, which normally radiate from the centrosome of the cell. They alternate between phases of growth and shortening by the addition or removal of tubulin subunits, primarily at the growing ‘plus’ end. Microtubules are active in a variety of cellular processes such as cell motility, cell division, intracellular transport, and in maintaining the spatial distribution of organelles [6,7]. The intermediate filaments are the most structurally and functionally diverse elements of the cytoskeleton, composed of neutral subunits of α-helical polypeptide chains, which often exhibit cell- or tissue-specific distributions and properties. Their varied composition permits intermediate filaments to respond differently to physical stress, facilitating force transmission from the plasma membrane to the nucleus [8]. The actin cytoskeleton occupies a variety of assembly formations to provide the basic framework for cell shape, motility and intracellular organization. Due to its diverse range of functions, actin has the intrinsic capability to assemble and disassemble filaments as required, most notably in the form of stress fibers [[9], and reviewed in [10]]. Bundles of aligned actin filaments also support filopodial protrusions, whereas networks of highly branched filaments support the leading edge in motile cells and generate the forces associated with changing cell shape [reviewed in [5]] (Figure 1). 

As a regulator of cell motility and due to its involvement in signal transduction pathways, the actin cytoskeleton frequently interacts with integrin proteins in the plasma membrane. Cell adhesion via integrins leads to focal adhesion formation through a cascade of cell attachment, cell spreading and actin reorganization. In their capacity as cell adhesion receptors, integrins physically anchor the cell and function in signal transduction across the cell membrane. A number of cytoskeletal linker proteins, such as talin, which possesses both actin and integrin binding sites, bind to the cytoplasmic tails of the integrin β subunits leading to receptor activation and focal adhesion protein recruitment [reviewed in [11,12,13]]. These complexes then develop into larger focal adhesions, which extend deeper into the cell away from the periphery, via a mechanism dependent on Rho GTPases. Focal adhesion complexes function as signaling centers ensuring substrate adhesion and targeted location of actin filaments and signaling components within the cell, rendering them essential for establishing cell migration and maintaining cell growth and survival [[14], reviewed in [15]].

Altogether, the dynamics of actin and focal adhesion remodeling represent foundation events central to overall cell health and function and generally microscopy techniques are well suited to the study of the actin cytoskeleton, particularly when deployed in living cells. Exploitation of fluorescence has made it possible to visualize and analyze complex dynamic cellular events at the organelle and sub-organelle level, allowing biochemical, biophysical, and spatio-temporal observations of the cell to be made [reviewed in [16]]. However, it must be noted that different cell types exhibit variations in the factors regulating actin filament organization and distribution [17], and focal adhesion composition, which themselves can also vary within a single cell [18]. Although both actin and focal adhesions share the same basic function and design in many cell types, subtle differences in their intracellular distribution may need to be accounted for when elucidating their function (Figure 1). In this review we highlight current practices, as well as a number of developing imaging modalities and emerging techniques in the field (Table 1), which provide new insight into how focal adhesions and the actin cytoskeleton are coordinated. 

**Table 1:** Examples of imaging techniques used to visualize and analyze the cytoskeleton and focal adhesions in cells.

Imaging Technique	Basic Principle	Advantages	Disadvantages	Reference
Laser scanning confocal microscopy (LSCM)	Out-of-focus light is eliminated from sample via a pinhole	Relatively straightforward to use, optical sectioning allows imaging of thin optical slices in thick samples	Photobleaching, slow acquisition not well suited for living cells	[20,24]
Spinning disk confocal microscopy (SDCM)	Illumination via multiple pinholes	Rapid acquisition with minimal illumination of specimen, ideal for live-cell imaging	Pinhole crosstalk increases background signal	[23]
Interferometric photoactivated localization microscopy (iPALM)	Combines photoactivated localization microscopy with single-photon, simultaneous multiphase interferometry	Images a high density of specific fluorescent molecules with 3D nanoscale (10-20 nm) resolution	Limited by specimen thickness, complexity of microscope setup	[30]
Electron cryotomography (cryo-ET)	Electrons are used to produce projections of a sample at multiple angles which are back-projected to reconstruct the original object in 3D	Rapid freezing preserves sample in near-native state without need for fixation or staining	Limited number of images can be acquired due to the target area becoming electron irradiated	[26]
Scanning angle interference microscopy (SAIM)	Modified FLIC microscopy which actively scans the incidence angle of excitation	Provides nanometer precision and allows temporal sampling rates per second	Unknown refractive indices of sample structures cause minor errors in determining precise absolute height measurements	[31]
High content screening (HCS)	Automated fluorescence microscopy (confocal or non-confocal)	Quantitative cellular imaging producing large data sets in a relatively short time period	Lack of visual precision by researcher, potential issues with object segmentation during analysis	[43,44,45,51]

## 2. Confocal Fluorescence Microscopy

All cellular processes require the correct number of proteins to be assembled at particular times and in specific locations in the cell. Imaging technologies provide both an appropriate and powerful strategy to determine these characteristics, and in turn advance our current knowledge of the cytoskeleton and its diverse roles in the cell. Fluorescent labeling and visualization of proteins and lipids can be used with a wide range of imaging modalities, and offers high sensitivity, specificity, and importantly capability for quantification. Furthermore, when used in living cells, fluorescent labeling allows real-time visualization of protein localization, movement, and turnover. Due to the highly discrete nature of how the cytoskeleton is arranged, confocal microscopy has been a frequently exploited technique in its study. At the heart of the laser scanning confocal microscope (LSCM) is the pinhole, which functions to eradicate much of the ‘noise’ from out-of-focus light that can be observed in wide-field microscopy. Confocal microscopes therefore permit optical sectioning, allowing visualization of distinct slices from within relatively thick samples, which can then be converted into 3-dimensional (3D) reconstructions. In this way, spatial information can be derived, such as the distribution of a particular protein within a cell, or better yet, several proteins and their relationship with one another [reviewed in [19]]. Quantification of confocal image data using line scan analyses provides a convenient method to derive morphology information and study the distribution patterns of multiple proteins in parallel. Such an approach has recently been applied in mouse platelets fluorescently labeled for both actin and tubulin, allowing internal platelet contraction events to be quantitatively measured [20]. Stimulating the platelets with thrombin and treating with various drugs such as cytochalasin D, nocodazole and taxol, to either interfere with actin polymerization or microtubule dynamics, revealed a fundamental necessity of the actin cytoskeleton to impart platelet contraction following activation, with microtubules only partially governing the contraction process. 

Such LSCM studies are highly informative in terms of linking cytoskeleton protein location to function, however suffer from the key limitation that image acquisition is inherently slow, and therefore not compatible for observing cellular processes that occur in fractions of a second. This speed disadvantage has been overcome by the now widespread use of spinning disk confocal microscopy (SDCM), which generates confocality by using disks with multiple pinholes to simultaneously illuminate and collect light from across the entire sample but in a highly time resolved manner [reviewed in [21,22]]. This is particularly suited to studying the rapid dynamics of actin filaments and focal adhesion complexes at the plasma membrane, where excitation wavelengths for multiple fluorophores representing these two structures can be rapidly switched within microseconds [reviewed in [21]]. Recently, SDCM has been put to effective use in the study of dynamic actin filament remodeling during epithelial-mesenchymal transition (EMT) in mammary epithelial cells. The processes of EMT and cytoskeletal remodeling are dependent on an up-regulation in the expression of moesin, an actin-binding protein related to the regulation of cell adhesion, morphology, and migration via its role in linking the cytoskeleton to the plasma membrane. Epithelial cells exhibit thin cortical actin filament bundles, whereas in trans-differentiated mesenchymal cells they are reorganized into thick, parallel, contractile bundles, as demonstrated by one recent study [23]. Using fluorescence SDCM to monitor actin filament dynamics in live cells undergoing EMT, revealed a slow and progressive increase in the number and size of actin filaments during changes in cell morphology. Increased moesin expression promoted EMT and it is proposed that the transcriptional program driving this process controls progressive remodeling of actin filament architectures, as opposed to a rapid switch in actin filament organization.

Focal adhesion dynamics on the other hand can be regulated by tension imparted by actin reorganization. Photobleaching assays combining simultaneous fluorescence loss in photobleaching (FLIP) and fluorescence recovery after photobleaching (FRAP) were used to determine the kinetics of focal adhesion proteins. Combined with a nocodazole assay to disrupt the microtubule network, the FRAP-FLIP approach showed that microtubule disruption increased contractility within the cell via myosin light chain phosphorylation and increased actin filament accumulation. At small focal adhesions in the cell periphery, focal adhesion kinase (FAK) dynamics reduced while paxillin dynamics accelerated, but in contrast centrally located mature adhesions associated with actin exhibited more rapid FAK dynamics. Generation of a schematic cell model, based on z-scan images of living cells as an average indicator of shape, helped further analyze experimental FRAP-FLIP data for adhesion dynamics. Characteristic focal adhesion protein residence times were identified, with paxillin being found to remain in the adhesion twice as long as FAK. This application of fluorescence imaging and computer simulation technologies could lead to the development of a quantitative mapping technique for determining the residence times of all major focal adhesion and associated proteins based on their localization and migratory patterns [24]. 

## 3. Combined Imaging and Modeling Approaches

Heterogeneity of cell size, shape and protein distribution can be an issue arising from the use of fluorescence imaging data. However, to a large extent this may be overcome by computer modeling, which can accurately take into account the natural variations seen between cells and therefore across a set of fluorescence microscopy images. All-atom molecular dynamics simulations have been used to investigate the structure, properties and dynamics of the actin cytoskeleton based on a model fitted to reconstructions from electron tomograms (ET) [25,26]. Cryo-ET employs tomography to produce a 3D reconstruction of a sample based on tilted 2D images at cryogenic temperature and can represent complex biological structures at sub-nanometer resolution. The study by Pfaendtner and colleagues [25] specifically used cryo-ET images to focus on the actin filament branch junction formed by the Arp2/3 complex. Simulation data highlighted the presence of numerous salt bridges and hydrophobic contacts located at the interface between the Arp2/3 complex and a ‘mother’ actin filament, many of which exhibited dynamic properties over the timescale of the simulation through a combination of both formation and breakdown events. Analysis of the simulation data alongside the primary experimental data identified areas where the simulation provided atomic detail of the model structures. Computer modeling is advantageous in that it can hypothetically test infinite scenarios in a relatively fast manner compared to experimental approaches. Modern imaging techniques can contribute to simulation design based on information that can be extracted from imaging data. Using this approach, simulations can be modified and rapidly tested to predict potential outcomes, which if promising can then in turn be tested and verified by experimental means. Another application for such a technique is to predict migration patterns in cancer cells. An integrative cell migration model to predict the migratory behavior of cells on 3D curved surfaces was recently inspired by a set of 3D microfluidic migration experiments. Angiogenic sprouting of human microvascular endothelial cells was promoted by exposure to vascular endothelial growth factor (VEGF). Encompassing cytoskeletal and focal adhesion dynamics, actin motor activity and nucleus remodeling, the cell migration model indicated that migration velocity depends on the size of the channel through which the cells migrate [27]. It is hoped this model will aid in the design of future experiments for spontaneous cancer cell migration diagnostic assay development as well as experiments to further validate the model, which can be modified to incorporate wider mechanisms in cell biology such as cell-cell interactions.

## 4. Nanoscale Imaging

We now have a good knowledge of the molecular organization of focal adhesions [reviewed in [28]], but a large amount of these data derive from studies of *in vitro* protein–protein interactions. The mechanical properties of cells on particular surfaces or substrates are partially determined by the distribution of their focal adhesions. In order to define focal adhesion molecular architecture, an attempt to map the nanoscale organization of focal adhesion proteins has recently been described [29], using a super-resolution fluorescence technique, namely interferometric photoactivated localization microscopy (iPALM) [30]. This microscopy technique combines PALM with simultaneous multi-phase interferometry of photons from each fluorescent molecule, enabling imaging of a high density of specific fluorescently-tagged molecules with 3D nanoscale resolution. Imaging probes composed of photoactivatable fluorescent proteins fused to various focal adhesion proteins, including paxillin, vinculin and zyxin, were expressed in a human osteosarcoma cell line and mouse embryonic fibroblasts. The vertical distance separating integrins and actin was measured and the composition of the intervening region was identified. It was found to consist of defined protein layers. Specifically, an integrin signaling layer juxtaposed to the plasma membrane, a layer of FAK and paxillin, a layer containing talin and vinculin to facilitate force transduction, and an actin-regulatory layer containing zyxin and α-actinin. This work also established the consistency in the vertical distribution of the various focal adhesion components across a diverse range of adhesion sizes and shapes in various cell types. This suggests that the observed stratification of focal adhesion proteins is not cell-specific but rather a general organizing principle of these subcellular cellular structures.

Nanoscale imaging of protein dynamics in living cells remains a challenge but the development of new approaches based on currently used imaging techniques are beginning to address this issue. Paszek and colleagues [31] recently described a modified approach based on fluorescence interference contrast microscopy (FLIC), wherein the vertical position of nanometer-sized objects were determined using axially varying structured illumination. Rapid movements of proteins and their spatial organization can be captured by FLIC but this modified approach, termed scanning angle interference microscopy, allowed determination of protein positions over a broad axial range, resulting in images with axial precision at the nano level. This technique can identify distinct topographical features in fixed cells including focal adhesions, cortical actin and the downward bending of lamellar microtubules. However, in live cells this imaging platform is capable of acting as a molecular ruler providing spatial measurements of actin-associated proteins in focal adhesion complexes. The spatial positions of both the N- and C-termini of talin were identified, as well as the stratified position of paxillin and vinculin in motile cell adhesions. Scanning angle interference microscopy therefore shows great promise in identifying molecular structures at the nano level, but within timescales that allow for imaging of dynamic processes. It is expected that in the future this technique will be more widely applied in the study of other cellular processes, such as mechanotransduction, membrane transport, and cell motility.

## 5. Cell Migration Studies

The actin cytoskeleton and focal adhesion complexes also play a key role in cell migration. For example morphogenetic movements during embryonic development, the movement of neurites during development, chemotactic movement of immune cells, and fibroblast migration during wound healing are all facilitated by the actions of the cytoskeleton and focal adhesions. Cell migration essentially involves a cycle of four steps: protrusion of the leading edge of the cell, adhesion to a substrate, retraction of the rear of the cell, and detachment. The cell extensions that facilitate motility require the assembly of a specialized network of polarized actin filaments at the leading edge of the cell, and the adhesion step requires attachment via integrins and focal adhesion complex formation [reviewed in [32,33]]. However, collecting reliable quantitative data from migrating cells can be problematic in that there is high variability in parameters such as cell shape, protein localization and traction forces. Cytoskeletal organization varies greatly over time and between individual cells due to its dynamic behavior. Therefore, describing the overall organization of actin or the focal adhesions of cells can be problematic when conclusions are based on individual snapshot images. To address this problem, Keren and co-workers have exploited the natural phenotypic variability found in populations of motile epithelial keratinocytes to explore mechanisms of shape determination, and develop models relating cell geometry to cytoskeletal dynamics and membrane imposed forces based on quantitative observations [2]. By taking measurements of cell speed, area, and aspect ratio from a large number of live cells and correlating across the population, morphological information can be related to cellular actin dynamics. Tetramethylrhodamine-derivatised kabiramide C binds as a complex with G-actin to the free barbed ends of actin filaments, and was used to visualize actin filament distribution along the leading edge of the cell [34]. It was found that membrane tension imposed an opposing force on growing actin filaments, with force per filament being inversely proportional to local filament density. At the center of the leading edge, where filament density was high, membrane resistance was low, allowing filaments to grow rapidly and generate protrusions. At the rear of the cell, where the actin network disassembled, membrane tension effectively broke down the weakened network and moved actin filaments forward, resulting in cell retraction. This work therefore provides novel insight into how membrane tension and molecular processes occurring at opposite regions of the cell can be coupled, thus contributing to coordinated cell migration [2].

The concept of using ‘standardized’ cells has also been applied to study focal adhesion dynamics, by quantifying cell-substrate adhesion patterns in migrating keratinocytes through mapping against a standardized cell coordinate system [35]. Time lapse imaging of keratinocytes during cell migration was used to correlate areas of adhesion assembly, disassembly, and stability with actin flow and traction force patterns, ultimately discerning actin and focal adhesion dynamics. Each image frame was transferred to a cellular coordinate system and averaged, which revealed a polarized distribution of actin filaments and focal adhesions in migrating cells. The average actin signal was relatively low at the cell periphery but increased directly behind the nucleus where filaments accumulate into thick parallel bundles, forming contractile structures. Vinculin mainly localized at the cell periphery, forming an adhesion ridge along the cell border as density reduced toward the rear of the cell. The non-uniform distribution of focal adhesions demonstrates that the direction of movement of keratinocytes is dictated by regions of higher adhesion density and associated actin. Quantification of growth dynamics using the cellular coordinate system also revealed well-defined areas of adhesion assembly and disassembly, with assembling adhesions solely found at the leading edge, highlighting cell movement in the direction of newly formed and growing focal adhesions. Conversely, behind the leading edge there was a distinct shift toward disassembling adhesions, suggesting the breakdown of a substantial number of the newly formed focal adhesions that have left the leading edge. Together, these findings point toward a highly regulated pattern of focal adhesion dynamics directly related to the direction of migration. Furthermore, the methodology applied in this work provides an exciting new advance to the concept of the ‘standardized’ cell. Although many previous studies have employed micro-patterning of substrates with combinations of cell-repellent and cell-attractive materials to physically constrain cells into defined shapes, therefore facilitating their analysis [reviewed in [36], these substrates do not easily permit cell migration studies to be performed. Therefore, this modeling approach towards the ‘standardized’ cell opens up new avenues for studying cell migration and motility in real time, allowing visualization of potentially all the key cellular molecular machinery involved in the process. 

Combining imaging with advanced automated analysis algorithms can also prove useful in studying focal adhesions on an individual basis. This can provide precise detail of adhesion movement over time and critical information such as adhesion shape, size, number, location, and intensity from large populations of cells. Recently, Berginksi and colleagues [37] described a novel method to quantify focal adhesion dynamics in a migrating cell line (NIH 3T3 fibroblasts). Using images from a highly time-resolved total internal reflection fluorescence microscopy (TIRF) system, algorithms were developed to identify and track individual focal adhesions. Typically they analyzed between 1,000-10,000 adhesions per cell, based on EGFP-paxillin. Inhibition of cell motility by a single point mutation at the c-Jun N-terminal kinase phosphorylation site S178A site on paxillin affected focal adhesion assembly rate, size, and site formation, illustrating how this method can be applied to analyze a range of complex adhesion phenotypes as well as their entire lifespan. 

Force mechanics must also be considered during focal adhesion assembly, maturation, and disassembly in motile or migrating cells. The maturation stage is a mechanosensitive process, defined by the recruitment of in excess of 200 different proteins into the complex [12,38]. Geiger and Zaidel-Bar [39] describe a range of proteomics studies, which point toward a larger pool of focal adhesion associated proteins than are currently known, as well as refining current understanding of adhesion regulation. However, development of a calibrated biosensor that applies single molecule force spectroscopy to a protein has enabled the measurement of forces across single proteins (in this case vinculin) within cells, providing novel insight into force generation during adhesion mechanics [40]. Vinculin functions in connecting integrins to actin filaments via talin, and regulates mechanotransduction from the extracellular matrix to the actomyosin machinery. Activation and recruitment of vinculin to focal adhesions is force-dependent and through its interaction with the complex of integrin, talin, and actin, it facilitates focal adhesion composition by controlling the recruitment and release of core components [41,42]. Grashoff and colleagues have measured the tension across vinculin in a stable adhesion. It was noted that highest tensions occurred during adhesion assembly and enlargement, while lower force was exerted in disassembling adhesions of the trailing edge of migrating cells. A tension sensor module with an elastic domain was inserted between a pair of fluorophores that exhibit fluorescence resonance energy transfer (FRET), with the distance between them providing a readout of the amount of tension being exerted. Thus, a reduction in FRET was expected in response to tension. This was evident in the response to cellular forces, whereby cells genetically depleted of vinculin but containing the tension sensor module showed a reduced FRET index in focal adhesions formed on a fibronectin-coated surface. Fluorescence lifetime microscopy (FLIM) also revealed significantly longer lifetimes in these adherent cells, suggesting an escalation in mechanical tension. 

## 6. Automated High Content Screening Microscopy

This review has thus far highlighted several novel imaging-based approaches and analysis methods that have furthered our understanding of actin and focal adhesion dynamics in living cells. However, these studies generally take account of known molecular machinery, despite it being clear that further regulatory molecules are undoubtedly needed for cytoskeletal function, and remain to be identified. The development of small interfering RNA (siRNA) technology, particularly in combination with automated high content screening (HCS) microscopy—an imaging method to quantitatively describe cellular phenotypes from very large numbers of cells and perturbations—has paved the way to perform systematic down-regulation screens in mammalian cells. This approach is not only likely to be highly fruitful in identifying ‘missing’ machinery and regulatory molecules needed for cytoskeletal function, but also to reveal the interplay between the cytoskeleton and other cellular processes and events. A high throughput wound healing siRNA screen targeting over 1000 human genes was established to systematically analyze epithelial cell migration [43]. In order to implement this assay in a high throughput manner, a robotic-driven pin delivered a precise scratch in confluent cell monolayers. The set of siRNAs screened were obtained from three libraries; the human phosphatase and kinase libraries, and a custom library targeting genes with known or predicted roles in migration or adhesion. Three categories of candidate genes, or ‘hits’, were identified in this study; 34 genes with a negative regulatory role in migration, 32 with a positive role and 29 with an effect on cell proliferation. Amongst these hits, 42 had not previously been associated with cell motility or adhesion. This carefully conducted study revealed a limitation of protrusion formation in cells transfected with siRNAs affecting migration, suggesting that these genes influence actin cytoskeleton rearrangements associated with lamellar dynamics. This screen identified a large number of genes regulating cell migration, both positively and negatively, and highlights the relationship between enhanced cell migration and impaired cell-cell junctions, suggesting these genes as regulators of cell-cell adhesion. Stemming from the results of this study, Winograd-Katz and colleagues [44] conducted a screen of three siRNA libraries (1,180 siRNAs) aimed at migration- and adhesion-related genes as well as genes for protein and lipid kinases and phosphatases to assess focal adhesion morphology, distribution, and their influence on cell adhesion. Employing HeLa cells expressing YFP-paxillin, a high-throughput approach revealed a correlation effect between several morphological features of adhesions. These features included focal adhesion length and area, and paxillin intensity within the adhesion. The pairs of features that correlated highly occurred between focal adhesion area and length, between area and paxillin intensity, and between length and intensity. The authors proposed a model for hierarchical adhesion regulation based on these correlation observations and further identified specific gene clusters associated with the cytoskeleton that uncoupled previously identified correlations. More recently, an siRNA screen in *Drosophila* cells has been interrogated to provide additional candidates associated with actin filament organization, cell morphology and migration [45]. The rationale behind this latter screen was that by specifically looking for genes affecting cell shape and actin distribution, there is a lower probability of identifying indirect cell migration factors, for example genes associated with cell division, that potentially would still appear as positive hits in a wound healing screen. In this regard, this small-scale study was highly successful in identifying a total of 26 new regulators of cytoskeletal organization. 

**Figure 2:**
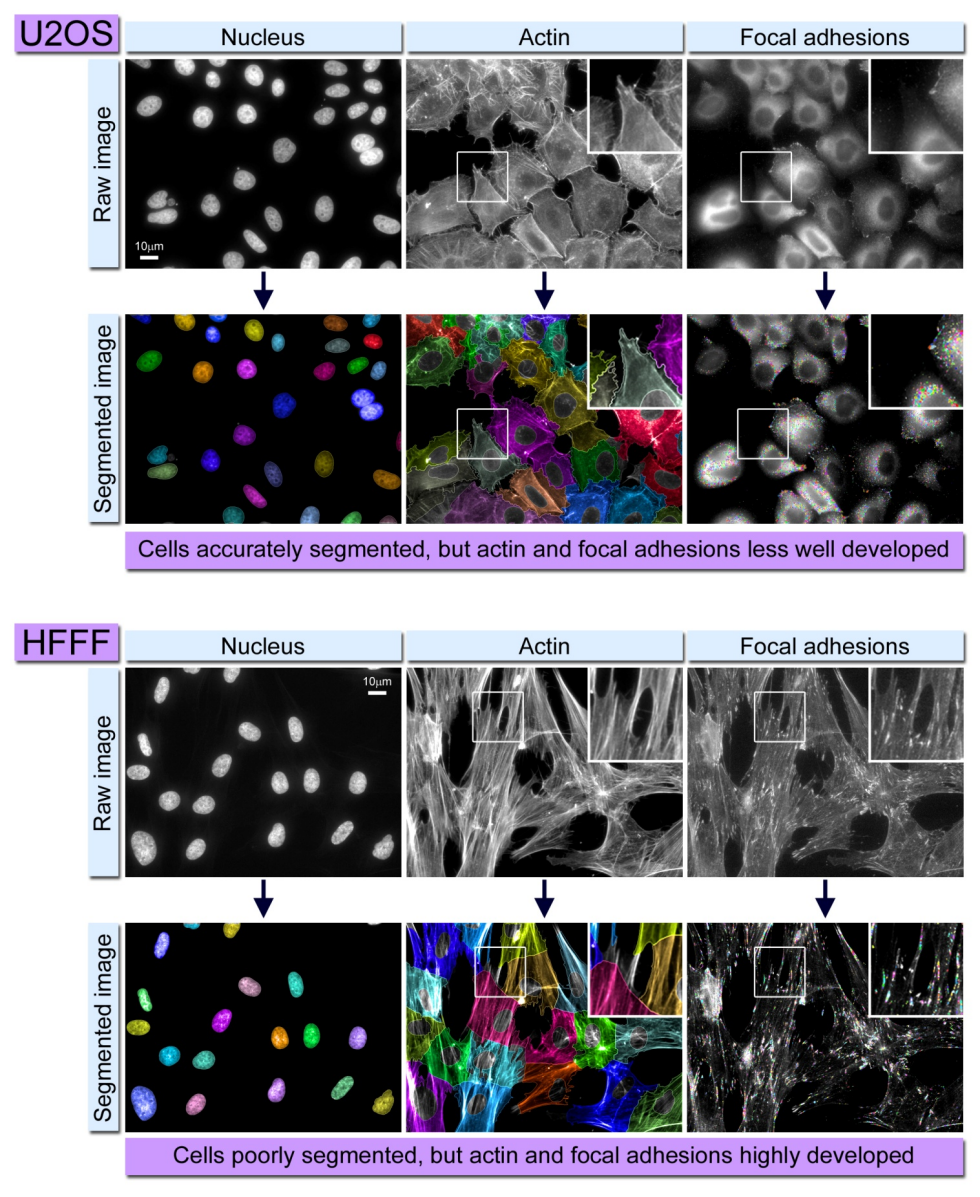
Example of problems that can arise during automated image analysis of cytoskeletal structures. Upper panel shows U2OS osteosarcoma cells fluorescently labeled for actin and focal adhesions. The cytoplasm of each individual cell (shown in the actin channels) is accurately segmented; however the focal adhesions are small and not prominent, therefore resulting in their poor detection (and therefore quantification) by the software. The lower panel shows HFFF (human fetal foreskin fibroblast) cells treated the same way as above. These particular cells have a well-developed cytoskeleton, and the focal adhesions are accurately identified. However as the peripheries of these cells grow over one another, their individual cytoplasms (and therefore cells) are often segmented incorrectly. In the processed (segmented) images the color coding is simply used to denote distinct objects (either nuclei, cytoplasms or individual focal adhesions).

Cell polarity is also governed by actin dynamics in conjunction with intracellular signaling cascades, and is associated with several cellular processes, including differentiation and proliferation. Disrupted regulation of cell polarity is linked to cancer as well as developmental disorders, thus in addition to screening for cell migration, combined siRNA and imaging approaches have also been put to effective use in identifying genes associated with cytoskeleton-driven cell polarity. In a study addressing the molecular mechanisms underlying fibroblast polarity on extracellular matrices of different rigidities, it was demonstrated that polarization of these cells requires rigid matrices, and that the mechanosensing machinery of focal adhesions aid in regulating the rigidity-dependent polarization process [46]. Fibroblast spreading on rigid surfaces proceeded with radial spreading, followed by cell polarization; the latter characterized by an increase in cell aspect ratio and stress fiber elongation along the major cell axis. Examination of focal adhesion formation on substrates of varying rigidity, using fibroblasts expressing paxillin-YFP, revealed major differences in focal adhesion morphology and dynamics. On rigid matrices, large and uniformly oriented focal adhesions formed along the major cell axis aligned to actin stress fibers, whereas small and radially oriented adhesions occurred in cells growing on substrates of reduced stiffness. However, it was also noted that focal adhesions on the rigid substrate were considerably less dynamic than those formed on a substrate of lower rigidity. Live cell monitoring revealed focal adhesion alignment occurring prior to overall elongation of the cell, suggesting that focal adhesion orientation could facilitate cell polarization. Depletion of a number of protein tyrosine kinases (PTKs) using siRNAs markedly altered focal adhesion formation, cell polarization and cell traction force generation. This ultimately suggests that different stages of cell polarization are regulated by several PTK-dependent molecular pathways, which mutually regulate cell contractility and focal adhesion-mediated mechanosensing. One drawback in the study of focal adhesion dynamics is the ability to reliably identify, segment and track changes occurring in the lifetime of an adhesion. Although some studies have achieved this successfully with relatively low sample (cell) numbers [39], automated imaging platforms as used in HCS typically acquire thousands of images containing hundreds of thousands of cells, meaning that it is impossible to manually quality control the acquisition and analysis. Nevertheless, if we are to fully catalogue the genes associated with particular cellular processes, we require automation and systematic processing of large data sets. In certain cell types, segmentation can be a particular challenge and therefore improvements to algorithms used for cell or focal adhesion segmentation must be addressed. Some of the pitfalls encountered with automated image analysis include cells growing in colonies or clusters, which tend to overlap making it difficult to identify their borders (See Figure 2 for examples). Furthermore, under these circumstances focal adhesion segmentation presents a challenge whereby adhesions in one cell may be identified as part of a neighboring cell in cases where cell borders are unclear. A number of recently described analysis tools and algorithms are now beginning to address such problems [47].

Finally, it is also worth noting that siRNA screening combined with advanced cell imaging techniques are now revealing wider cellular roles for the actin cytoskeleton and its regulatory machinery. For example, membrane dynamics and subcellular trafficking events in cells have long been associated with the microtubule network [reviewed in [48], [49]], but recently a key role has been highlighted for the actin machinery in these cellular processes. Live cell imaging in mouse oocytes, followed by automated vesicle tracking, indicated that GFP-Rab11-positive carriers emanating from the endosomal network critically require actin in order to traffic to the cell surface [50]. Furthermore, these long range transport vesicles have the ability to recruit actin nucleation factors from the spire and formin families, in order orchestrate this process. Work from our own laboratory has also identified actin regulatory proteins as playing a pivotal role in secretion, specifically in the early part of the pathway between the endoplasmic reticulum (ER) and the Golgi complex. Following a genome-wide siRNA screen for genes influencing general secretory pathway function, in depth analysis was carried out on 554 of the genes identified as ‘hits’. The link to actin cytoskeletal organization focused around a network of proteins; the GTPase regulator SOS2, the GTPase RhoU, and the GTPase activating proteins (GAPs) ARHGAP12 and ARHGAP44. Knockdown of these cytoskeleton regulatory proteins caused phenotypic effects in the early secretory pathway. Depletion of RhoU increased the number of stress fibers observed, whilst knockdown of the two GAPs altered cell morphology, led to a discernible loss of F-actin, and accumulation of actin in short disorganized bundles. However most strikingly, depletion of each of these proteins resulted in gross changes to the organization of ER exit sites, the specific membrane structures in which secretory proteins are first concentrated at the ER. The consequence of this was a corresponding reduction in secretory pathway function [51]. These findings serve to highlight the vastly integrated nature of mammalian cells, and that the actin cytoskeleton is likely to play a much wider cellular role than previously predicted.

## 7. Conclusions

The idiom “seeing is believing” cleverly encapsulates the significance of imaging in cell biology. Advances in various microscopy techniques and continuous developments in methodologies provide cell biologists with ever more powerful tools to dissect complex cellular processes. This is particularly relevant to the actin cytoskeleton, in which protein assemblies and dynamics are highly complex, requiring increasingly sophisticated solutions if we are to fully understand this intracellular network. Fluorescent protein technology has undoubtedly made a huge impact in this regard [reviewed in [52]], and its application from fundamental biological research through to biomedical use continues to increase. Traditionally the actin cytoskeleton has been visualized with chemical tools such as fluorescently-labeled phalloidin (Figure 1), which although are useful, have limited application in living cells. An increasing number of novel live cell-compatible probes are now being developed to counter the problem that direct fluorescent labeling of the actin polypeptide inhibits its function and dynamics. Such probes include the calponin homology domain of the actin-binding protein utrophin fused to GFP (and its variants) [53], a photo-activatable utrophin fusion construct [54], and the 17 amino acid peptide “LifeAct” (derived from the yeast actin-binding protein ABP140) [55]. Clearly their increased use in the context of the imaging technologies discussed here will further add to our understanding of cytoskeleton dynamics in living systems.

**Figure 3:**
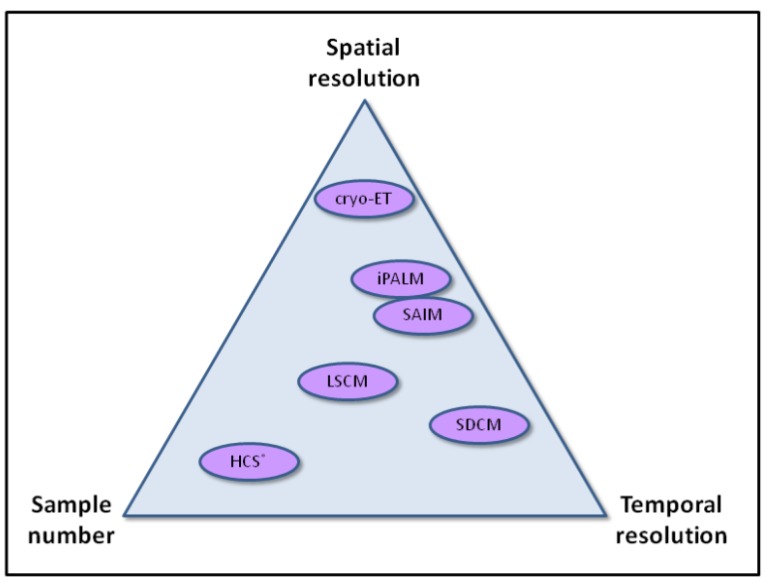
Schematic representation of how various imaging modalities compare in terms of spatial resolution, temporal resolution, and sample number.; note that the temporal resolution of HCS is largely determined by the number of samples analyzed in parallel.

Although resolution in fluorescence light microscopy still remains a limitation to cell biologists, advances in super-resolution techniques and molecular modeling approaches increasingly provide new possibilities to understand actin function in the context of the living cell. Temporal information is also proving to be critical, and it is interesting to note in the new techniques that we highlight in this review, that some provide additional spatial resolution, and others temporal resolution. Applying these techniques to very large sample numbers, without losing information quality (resolution), will be one key challenge to overcome (Figure 3). Imaging is also becoming ever more quantitative. HCS and automated analysis approaches provide single cell data relating to size, morphology, intensity, texture and spatial distribution of cells and subcellular structures, which are all important attributes that can quantitatively describe actin and focal adhesion behavior. When used in combination with systematic gene down-regulation, we now have the opportunity to not only appreciate the complete molecular complexity of the actin cytoskeleton, but also how it integrates with other cellular processes. Further integration of information from methods such as those described here will propel our knowledge of cytoskeletal biology forward, and ultimately this information will be invaluable to understanding cell health.
